# Immune-modulatory effects of Spindlin-1 inhibitors

**DOI:** 10.1093/cei/uxaf013

**Published:** 2025-06-13

**Authors:** Susanne Schiffmann, Marina Henke, Friedemann Weber, Michael J Parnham

**Affiliations:** Fraunhofer Institute for Translational Medicine and Pharmacology ITMP, Frankfurt am Main, Germany; Institute of Clinical Pharmacology, Goethe-University Hospital Frankfurt, Frankfurt/Main, Germany; Fraunhofer Institute for Translational Medicine and Pharmacology ITMP, Frankfurt am Main, Germany; Institute for Virology, FB10-Veterinary Medicine, Justus-Liebig University, Giessen, Germany; Fraunhofer Institute for Translational Medicine and Pharmacology ITMP, Frankfurt am Main, Germany

**Keywords:** immune modulation, Spindlin-1, A366, MS31, energy metabolism, cytokines

## Abstract

Spindlin-1, a multivalent epigenetic reader, is a new target for cancer therapy. Beside the anticancer effect, modulation of the recognition of methyl marks of histones may impact the immune system, which plays an important role in the anticancer strategy of the human organism. Two Spindlin-1 inhibitors (A366, MS31) were characterized to differentiate between drug and target-specific effects. We performed a comprehensive study regarding the influence of Spindlin-1 inhibition on various immune cells. A366 and MS31 showed immune cell type-dependent cytotoxicity with IC_50_ values in the ranges of 37–143 µM and 11–3122 µM, respectively, macrophages tending to be less susceptible than lymphocytes. A366 had only minor effects on M1 polarization, whereas MS31 shifted the M1 to a M2 phenotype, as shown by regulated cytokines and surface marker expression. Both A366 and MS31 weakened the polarization of predifferentiated M2 macrophages by reducing surface marker expression, cytokines, and inflammatory markers. A366 and MS31 had no effect on activation and energy metabolism of CD4^+^ T cells. Interestingly, 5 µM A366 and 2.5 µM MS31 clearly prevented B cell activation, as shown by reduced proliferation, plasmablast formation, and release of immunoglobulins A and G. Additionally, A366 increased energy metabolism in B cells. In conclusion, the inhibition of Spindlin-1 had only minor effects on polarization of macrophages and T cell proliferation but profoundly prevented B cell activation at low concentrations. This suggests that Spindlin-1 inhibitors, while mediating anticancerogenic effects, may also suppress the humoral immune response and increase infection risk.

## Teaser

We performed a comprehensive study regarding the influence of Spindlin-1 inhibition on various immune cells. The inhibition of Spindlin-1 had only minor effects on polarization of macrophages and T cell proliferation but profoundly prevented B cell activation at low concentrations.

## Graphical Abstract

**Graphical Abstract:**
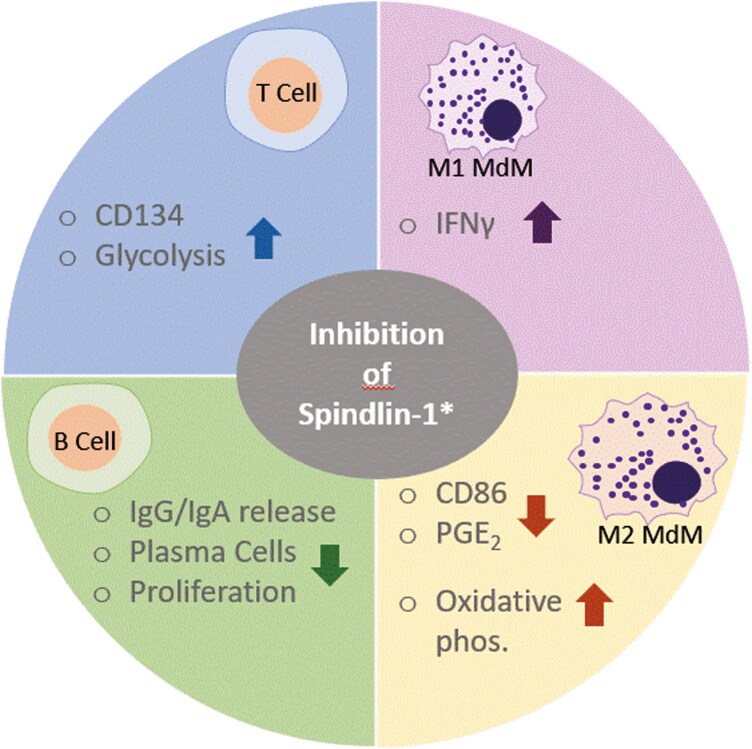

## Introduction

Histone posttranslational modifications rely on three types of proteins: enzymes that create (e.g. methyltransferases), remove (e.g. histone demethylases), or recognize (e.g. Spindlin-1) the modifications. Specifically, H3K9 methyltransferase (G9a) and G9a-like protein (GLP) are responsible for adding methyl groups to histone H3K9. This process results in chromatin inactivation and decreased expression of cancer suppressor genes, thereby promoting the growth and progression of various cancer types. Overexpression of G9a and GLP has been observed in lung, breast, bladder, colon, cervical, gastric, skin cancers, hepatocellular carcinoma, and hematological malignancies [1]. To target these cancer types, inhibitors of G9a and GLP, such as A366, have been developed. A366 has shown an IC_50_ of 3.3 and 38 nM for G9a and GLP, respectively [2], and also inhibits the histone reader protein Spindlin-1 [3].

There is increasing evidence suggesting that reader proteins also play a role in various human diseases, including cancer. Spindlin-1 is an epigenetic reader protein recognizing multiple histone methylation patterns leading to the activation of several cellular functions, such as ribosomal RNA transcription and cell cycle progression. Spindlin-1 recognizes H4K20me3, a marker in silenced heterochromatin regions, which might regulate DNA replication and cell cycle progression [4]. Moreover, it forms a complex with C11orf84 to recognize dual marks on histone H3 (K4me3 and K9me3). This recognition of bivalent histone marks leads to the relaxation of chromatin structure by dissociating heterochromatin protein 1 from condensed rDNA chromatin. As a result, RNA polymerase I can be recruited, enabling productive transcription of rRNA [5]. Hyperactive ribosome biogenesis is required to satisfy fast growth and proliferation of cancer cells [6]. Therefore, it is not surprising that Spindlin-1 is overexpressed in many types of human cancers and contributes to tumor cell growth, migration, and invasion [7, 8]. Knockdown of Spindlin-1 has been shown to suppress cell growth and proliferation in multiple tumor cell lines [9, 10]. A366 and MS31 are inhibitors of the Spindlin-1-H3K4me3-interaction (A366: IC_50_ = 72 nM; MS31: IC_50_ = 77 nM) and have been investigated for their antitumorigenic potential [11, 12].

Spindlin-1 is not only involved in the growth of tumor cells but may also play a role in the functioning of immune cells. It directly enhances expression of glial-derived neurotrophic factor (GDNF) an activator of the rearranged during transfection (RET) signaling pathway [13]. RET expression is similar across different subsets of immune cells, such as T cells, B cells, and monocytes [14]. RET controls IL-10 production in Th2 cells. Th cells deficient in RET show increased IL-10 production, while stimulation of Th1/2 cells with GDNF decreases IL-10 expression [15]. Additionally, there is a positive correlation between RET mRNA levels and interleukin 8 (IL8) mRNA levels, a cytokine produced by a variety of cells, including the adaptive immune cell types, neutrophils, monocytes, and macrophages [14]. These findings suggest that Spinlin-1 may have a functional role in modulating immune cell responses during inflammation and carcinogenesis through the RET signaling pathway.

In this study, we investigated the impact of inhibiting Spindlin-1 on cells of the immune system. We focused on analyzing different immune cell types, such as macrophages, CD4^+^ T cells, and B cells. To achieve this, two Spindlin-1 inhibitors, A366 and MS31, were used. Various aspects including surface marker expression, energy metabolism, cytokine release, and inflammatory mediator production in primary human immune cells were assessed. By using two different inhibitors, we aimed to distinguish between effects specific to the drug (A366, MS31) and effects specific to the target (Spindlin-1).

## Materials and methods

### Cells and reagents

Human primary B cells were cultured in RPMI 1640 medium supplemented with 2 mM glutamine, 0.05 mM ß-mercaptoethanol, 1 % penicillin/streptomycin 10 % FCS at 37 °C in 5 % CO_2_ atmosphere. Human primary CD4^+^ T cells were cultured in RPMI 1640 medium supplemented with 2 mM glutamine, 1 % penicillin/streptomycin 10 % FCS at 37 °C in 5 % CO_2_ atmosphere. Human primary monocytes and MdMs were cultured in RPMI 1640-Glutamax medium supplemented with 1 % penicillin/streptomycin 10 % FCS at 37 °C in 5 % CO_2_ atmosphere. RPMI 1640-Glutamax, RPMI 1640 medium, and glutamine were obtained from Gibco (Thermo Fisher Scientific, Oberhausen, Germany). Buffy coats from healthy donors were obtained freshly from DRK-Blutspendedienst (Frankfurt am Main, Germany). Orangu™ assay was purchased from Cell guidance systems (Cambridge, UK). Human FcR Blocking Reagent, human CD14 MicroBeads, human CD4 MicroBeads, human B cell isolation kit II, human granulocyte macrophage colony-stimulating factor (GM-CSF), macrophage colony-stimulating factor (M-CSF), bovine serum albumin, IL4 and all antibodies (except CD54) for surface staining were purchased from Miltenyi Biotec (Bergisch Gladbach, Germany). Anti-CD54 and CCL17 ELISA were obtained from Biolegend (Fell, Germany). Cytometric bead arrays for IFNγ, IL10, IL17, CCL2, IL8, and IL6 were purchased from BD Biosciences (Heidelberg, Germany). ELISA for CCL18 was purchased from BosterBio (Pleasanton, CA, USA). PGE_2_ ELISA was purchased from Enzo Life Sciences (Lörrach, Germany). Accutase® solution was obtained from Merck (Darmstadt, Germany). BioColl Trennlösung® was purchased from Bio&Sell (Feucht, Germany). EDTA was ordered from Sigma-Aldrich (Schnellendorf, Germany). sDC40L was obtained from Peprotech (Hamburg, Germany). CpG ODN 2395 was purchased from InvivoGen (Toulouse, France). Rapamacin and unconjucated goat antihuman IgM F(ab‘)2 fragments were ordered from Biomol GmbH (Hamburg, Germany). IgG ELISA, IgA ELISA, and Cell Trace violet were purchased from Thermo Fisher Scientific (Oberhausen, Germany). A366 and MS31 were purchased from MedChemExpress (Sollentuna, Sweden) and were dissolved in DMSO at a concentration of 100 mM and stored at −80 °C.

### Isolation of human primary cells

Human peripheral blood mononuclear cells (PBMC) were isolated from fresh buffy coats by density gradient centrifugation as previously described [16]. The quality of the buffy coats, specifically the amount of isolated PBMCs, varied; sometimes additional erythrocyte lysis was required. Buffy coats that resulted in a low PBMC count were not used for experiments. CD14^+^ and CD4^+^ cells were isolated with magnetic cell sorting using CD14 or CD4 MicroBeads from Miltenyi Biotec (Bergisch Gladbach, Germany) according to the protocol. B cells were isolated with the B cell isolation kit II (Miltenyi Biotec, Bergisch Gladbach, Germany) according to the protocol. The PBMCs were stained with a biotin-antibody cocktail consisting of biotin-conjugated monoclonal antibodies targeting CD2, CD14, CD16, CD36, CD43, and CD235a. Using antibiotin microbeads, the labeled cells were retained in the column via a magnetic field, while the B cell-enriched fraction was collected in the flow-through. The cell counts were determined using MACSQuant® Analyzer 10.

### Differentiation and polarization of human macrophages

The generation of monocyte-derived macrophages (MdMs) was performed as previously described [17]. Briefly, 0.5 × 10^6^ CD14^+^ cells/well were differentiated to macrophages in the presence of 10 ng/ml M-CSF (for M2 macrophages) or 10 ng/ml GM-CSF (for M1 macrophages) for 7 days. The macrophages were polarized in the presence of different concentrations of A366, MS31, or vehicle (DMSO) in 48-well plates. After pre-incubation with test substances or DMSO for 30 min, stimulants for differentiation were added: 20 ng/ml IFNγ for M1 macrophages and 10 ng/ml IL4 for M2 macrophages and incubated for 48h and 24h, respectively.

### Activation of CD4^+^ T cells and B cells

1.75*10^5^ CD4^+^ T cells (stained with 0.2 µM CellTrace^TM^ violet (CTV) (ThermoFisher Scientific, Schwerte, Germany)) pretreated with A366, MS31, or vehicle (DMSO) for 30 min in RPMI 1640 medium supplemented with 2 mM glutamine, 50 Units/ml IL-2, 1 % penicillin/streptomycin and 10 % FCS were seeded in anti-CD3-coated 96 well plates and stimulated with 2 µg/ml CD28 for 5 days. CTV staining was performed according to the protocol of supplier.

B cells were activated as described previously [16]. Briefly, 1.25 × 10^5^ B cells (stained with 0.25 µM CTV) per well were seeded in a 48 well plate and pretreated with A366, MS31, rapamycin, or vehicle (DMSO) for 30 min and then stimulated with 2.5 μg/ml CpG, 5 μg/ml unconjugated goat antihuman IgM F(ab′)2 fragments, 1 μg/ml sCD40L, and 50 ng/ml recombinant human IL-21 for 5 days.

### Viability assay

2 × 10^5^ CD4^+^ T cells, 1 × 10^5^ B cells, 1 × 10^5^ monocytes, 1 × 10^5^ M1 MdMs, or 1 × 10^5^ M2 MdMs were treated with A366, MS31, or vehicle for 48 h. The viability was determined with the Orangu^TM^ assay as described by the manufacturer.

### Surface marker detection and proliferation assay by flow cytometry

For analysis of the surface markers, 1.5–2*10^5^ cells of each sample were blocked with human FcR Blocking Reagent (15 min, 4 °C). Afterwards, samples were stained with up to 7 µl of a mixture of different surface marker antibodies (15 min, 4 °C) and 250 µl of 10 % FCS/PBS were added. After centrifugation (300 g, 5 min, 4 °C) samples were resuspended in 100 µl PBS and measured with MACSQuant® Analyzer 10 flow cytometer. For M1-/M2-MdMs, anti-CD86, anti-CD14, anti-HLA-DR, anti-CD206, anti-CD80, anti-TREM2, and anti-CD163 were used. For CD4^+^ T cells, anti-CD69, anti-CD45RO, anti-CD134, anti-CD154, anti-CD3, anti-CD25 were employed. For B cells, anti-CD27, anti-CD19, and anti-CD38 were applied. Zombie Aqua™ Fixable Viability Kit (1:500 dilution, BioLegend, San Diego, CA, USA) (for CD4^+^ T cells, macrophages) or Annexin/PE staining (BD Biosciences, Heidelberg, Germany) (for B cells) was used to discriminate living and dead cells according to the manufacturers protocol. For T cell and B cell proliferation assays, freshly isolated T and B cells were stained with CTV and the decrease in CTV as a result of cell proliferation was detected by flow cytometry. CTV positive cells were gated in high proliferating cells (low CTV level), medium proliferating cells (medium CTV level), and nonproliferating cells (high CTV level). Plasmablasts were characterized as CD27^+^CD38^+^ cells, naïve B as CD19^+^CD27^low^CD38^med^, and memory B cells as CD19^+^CD27^med^ CD38^low^ (Supplementary Fig. 1). For M1 MdMs, the cell population was gated using FSC/SSC channel, for M2 MdMs, the CD163^med^ population was gated since IL4 downregulates CD163 [18] and for CD4^+^ T cells, CD3^+^CD25^+^ cells were gated. Geometric mean of the fluorescent intensity was calculated using FlowJo software V10 (Treestar, Ashland, TN, USA). Fold induction of surface marker expression was calculated using the DMSO-treated cells as control.

### Cytokine, Ig, and PGE_2_ detection by cytometric bead array or ELISA

For determination of cytokine concentrations (IL10, IL17, IL8, IL6, IFNγ, CCL2) in the supernatant of humane immune cells, cytometric bead array (BD Biosciences, Heidelberg, Germany) was used. For the detection of IgG, IgA, PGE_2_, CCL18, and CCL17 an ELISA was performed. The cytometric bead arrays and the ELISAs were performed according to the manufacturers protocol.

### Determination of energy metabolism

The energy metabolism was determined as described previously [17]. Briefly, for the analysis of the extracellular acidification rate (ECAR) and the oxygen consumption rate (OCR) for human M1 MdMs, M2 MdMs, and activated B-/ CD4^+^ T cells, the Seahorse XFe96 FluxPak (Agilent, Waldbronn, Germany) was used as recommended by the supplier. MdMs were polarized to M1 or M2 MdMs, B- and CD4^+^ T cells were activated in the presence of A366, MS31, or vehicle (DMSO) for 48 h (M1 MdMs), 24 h (M2 MdMs) or 5 days (T/B cells). Cells were washed with Seahorse XF RPMI medium pH 7.4 (Agilent, Waldbronn, Germany), and OCR and ECAR were measured for a period of 120 min using the Seahorse XFe96 Analyzer (Agilent, Waldbronn, Germany) and analyzed by Wave Software (Agilent, Waldbronn, Germany). For T/B cells, the values after 50 min of stimulation of samples were related to the unstimulated control sample. For macrophages, the values at 50 min in treated samples were related to the vehicle control.

### Statistics

Results are presented as means ± standard errors (SEM). For each biological replicate (n) three technical replicates were performed. For all calculations and creation of graphs, GraphPad Prism 9.2.0 was used and *P* < 0.05 was considered as the threshold for significance. The statistical analysis applied is denoted in the figure legends. Flow cytometry data were analyzed by FlowJo Verison 10.5.3.

## Results

### Spindlin-1 modulators mediate cell type-dependent cytotoxic effects

**Figure 1:**
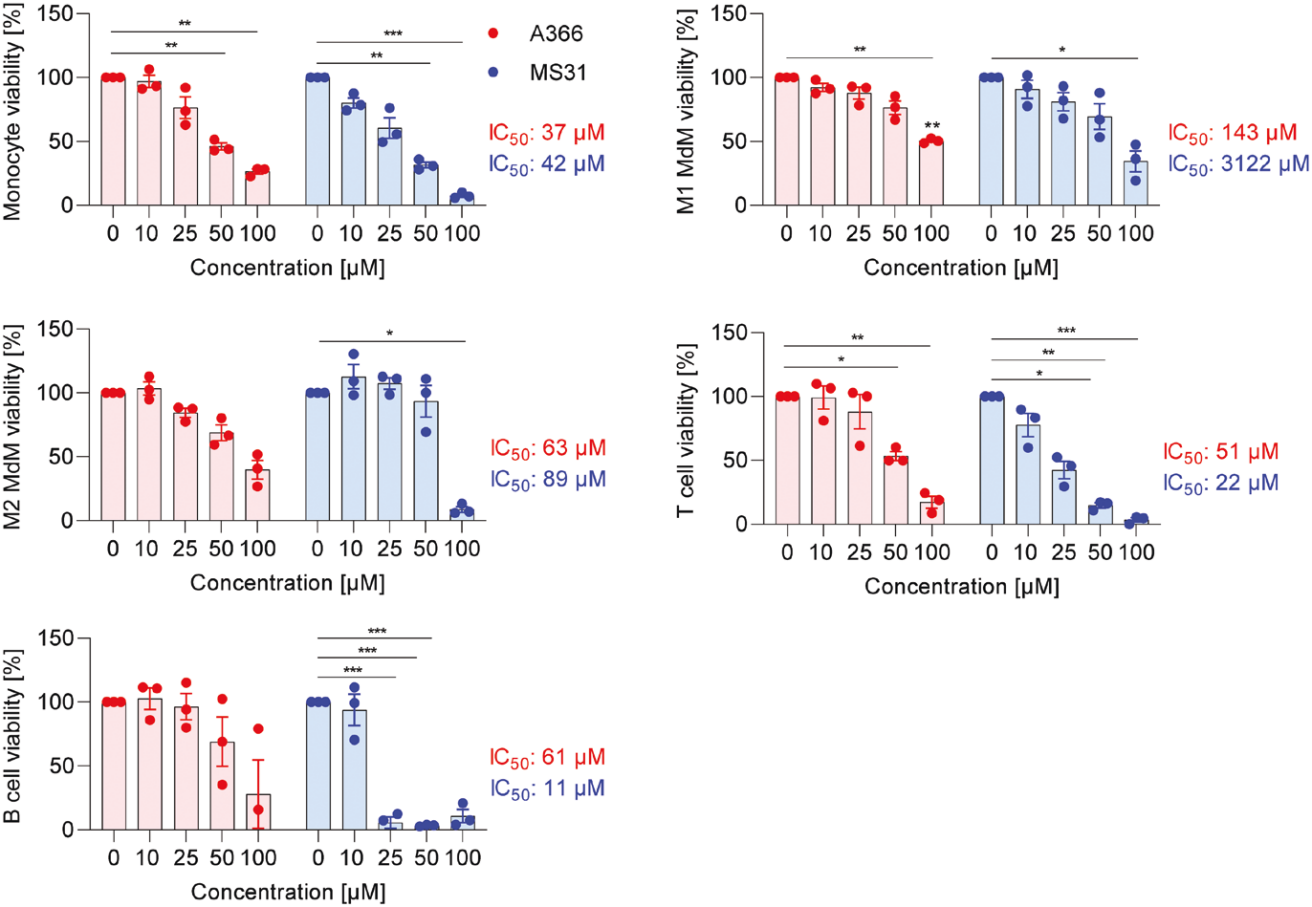
effect of Spindlin-1 modulators on immune cell viability. Monocytes, M1 MdM, M2 MdM, CD4^+^ T cells, and B cells were treated with A366, MS31, or vehicle in the indicated concentrations for 48 h. Viability was determined by a formazan-based assay. The viability values of treated samples were related to vehicle. *n* = 3. Two-way ANOVA with Sidak`s multiple comparisons was used to determine significant differences between treated and control samples. A nonlinear fitting model was used for the calculation of the IC_50_ values using GraphPad Prism 9.2.0. *P < 0.05, **P < 0.01, ***P < 0.001

First, we tested the effect of Spindlin-1 modulators on the viability of primary human immune cells to identify nontoxic concentrations that could be used in the functional assays. We treated monocytes, polarized MdMs (M1/M2), CD4^+^ T cells, and B cells with different concentrations of A366, MS31, or vehicle for 48 h and determined viability with a formazan-based assay. For A366, the IC_50_ values for CD4^+^ T cells, B cells, monocytes, and M2 MdMs ranged from 40 to 80 µM. However, M1 MdMs showed less susceptibility to A366, with IC_50_ values of approximately 140 µM (Fig. 1). Interestingly, MS31 exhibited higher cytotoxicity than A366 for CD4^+^ T cells and B cells, with IC_50_ values ranging from 11 to 22 µM. For macrophages (M2 MdM, M1 MdM), the IC_50_ values for MS31 were higher compared to A366 (Fig. 1). Only for monocytes were the calculated IC_50_ values within the same range for both A366 and MS31. In conclusion, these findings indicate that different concentration ranges of MS31 and A366 need to be used for subsequent functional assays depending on the cell types involved.

### Spindlin-1 inhibitors affect polarization of macrophages

**Figure 2:**
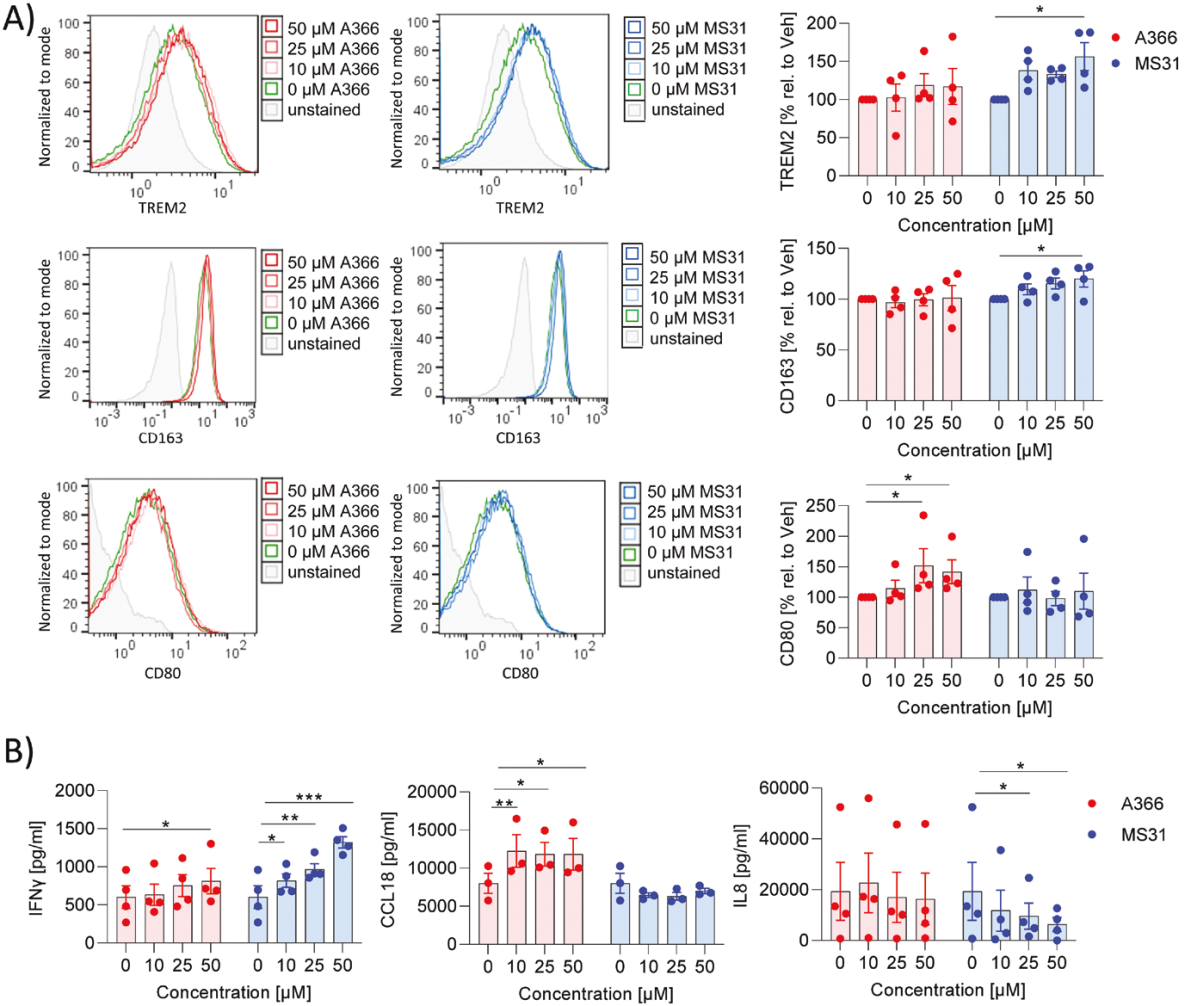
effect of Spindlin-1 modulators on M1 polarization. Monocytes isolated from buffy coats by magnetic cell sorting were differentiated to macrophages by the addition of 10 ng/ml GM-CSF. Macrophages were polarized with 10 ng/ml IFNγ to M1 macrophages in presence or absence of A366 or MS31 with the indicated concentrations for 48 h. (a) Surface markers were determined by flow cytometry. MFI values (left panel) and % values (right panel) are shown. To obtain the % values the MFI values of treated samples were related to vehicle. (b) Cytokines were determined by cytometric bead array or ELISA in the supernatant. n = 3. Two-way ANOVA with Dunnett`s multiple comparisons was used to determine significant differences between treated and control samples. *P < 0.05, **P < 0.01, ***P < 0.001.

Spindlin-1 inhibitors are being studied for their potential therapeutic applications, particularly in cancer treatment, as Spindlin-1 is known to be overexpressed in various cancer types [7]. Since cancer drugs often exert immune-suppressive effects, we were interested to know if Spindlin-1 inhibitors also modulate the immune response. Firstly, we investigated whether the inhibition of Spindlin-1 influences the polarization of MdMs to M1 or M2 MdMs. During the polarization of M1 macrophages, the concentrations of A366 (0–50 µM) and MS31 (0–50 µM) used did not induce cytotoxicity (Supplementary Fig. 2a). Also, various pro- (CD80, CD86, HLA-DR) and antiinflammatory/resolving (TREM2, CD206, CD163) surface markers were analyzed by flow cytometry. A366 induced minor upregulation of CD80, whereas MS31 slightly increased CD163 and TREM2 expression (Fig. 2a). The other surface markers were not influenced (Supplementary Fig. 3a). Since the release of cytokines and inflammatory mediators is a main player in the immune response, we analyzed several of these markers (CCL2, CCL18, CCL17, IL8, IL6, IFNγ, PGE_2_). A366 significantly increased the release of IFNγ and CCL18, whereas MS31 upregulated the release of IFNγ more pronouncedly and significantly inhibited the release of IL8 (Fig. 2b; Supplementary Fig. 3b). These data indicate that A366 has only minor effects on M1 polarization, whereas MS31 may shift M1 to an M2 phenotype.

**Figure 3:**
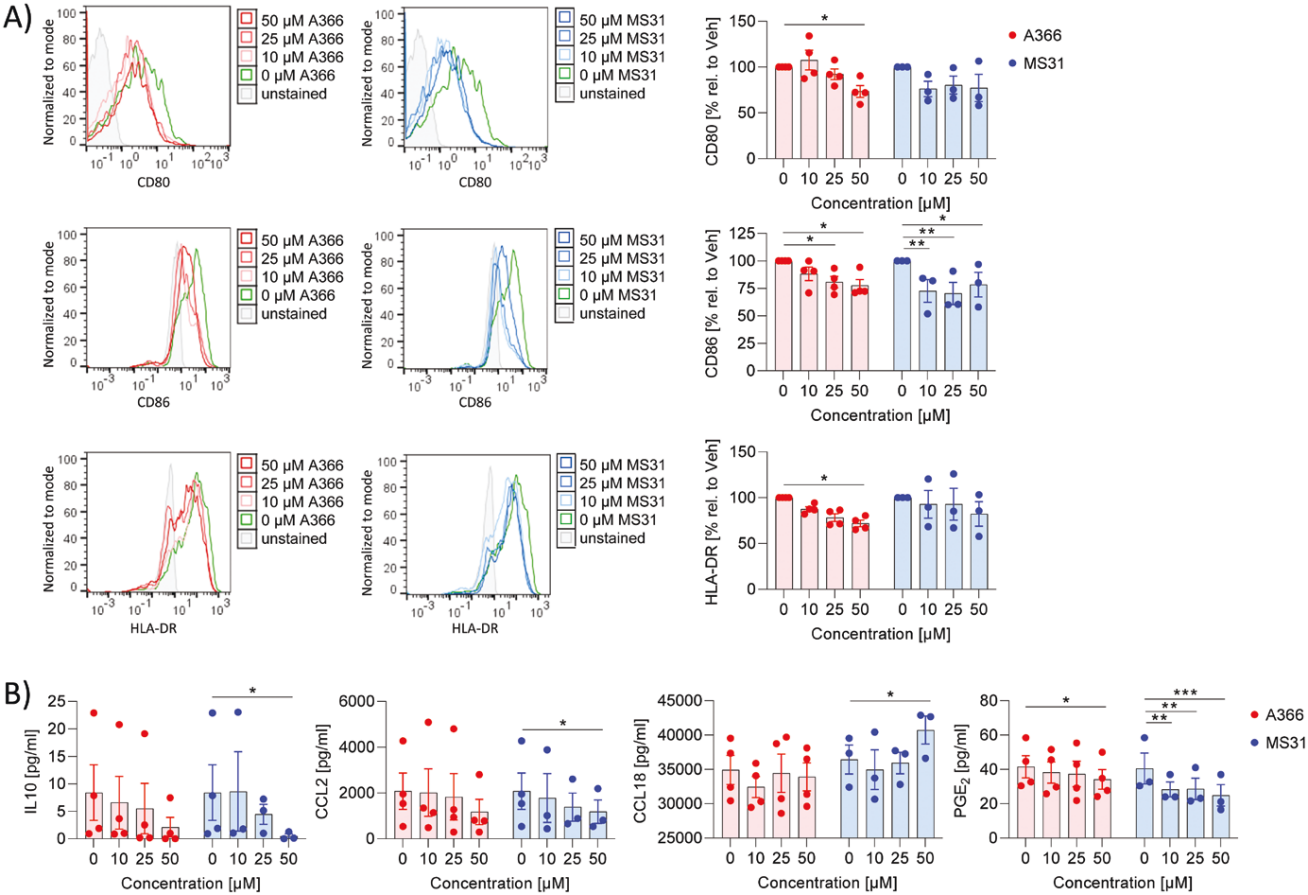
effect of Spindlin-1 modulators on M2 polarization. Monocytes isolated from buffy coats by magnetic cell sorting were differentiated to macrophages by the addition of 10 ng/ml M-CSF. Macrophages were polarized with 10 ng/ml IL4 to M2 macrophages in presence or absence of A366 or MS31 with the indicated concentrations for 24 h. (a) Surface markers were determined by flow cytometry. MFI values (left panel) and % values (right panel) are shown. To obtain the % values the MFI values of treated samples were related to vehicle. (b) Cytokines were detected by cytometric bead array or ELISA in the supernatant. *n* = 3–4. Two-way ANOVA with Dunnett`s multiple comparisons was used to determine significant differences between treated and control samples. *P < 0.05, **P < 0.01, ***P < 0.001.

A366 and MS31 present during the polarization of macrophages to M2 MdMs did not induce cytotoxicity over the concentration range tested (0–50 µM) (Supplementary Fig. 2b). A366 reduced slightly anti- (TREM2, CD206) and more pronounced pro-inflammatory (CD80, CD86, HLA-DR) surface marker expression, whereas MS31 reduced CD86 expression (Fig. 3a; Supplementary Fig. 4a). The effects of Spindlin-1 inhibitors on the release of inflammatory markers were limited. A366 reduced slightly PGE_2_ release and MS31 reduced PGE_2_, IL10, and CCL2 release and increased CCL18 (Fig. 3b). IL8 and CCL17 were not influenced (Supplementary Fig. 4b). Due to the downregulation of several inflammatory markers, these data indicate that A366 and MS31 may weaken M2 polarization.

### Spindlin-1 inhibitors reduced glycolysis

**Figure 4:**
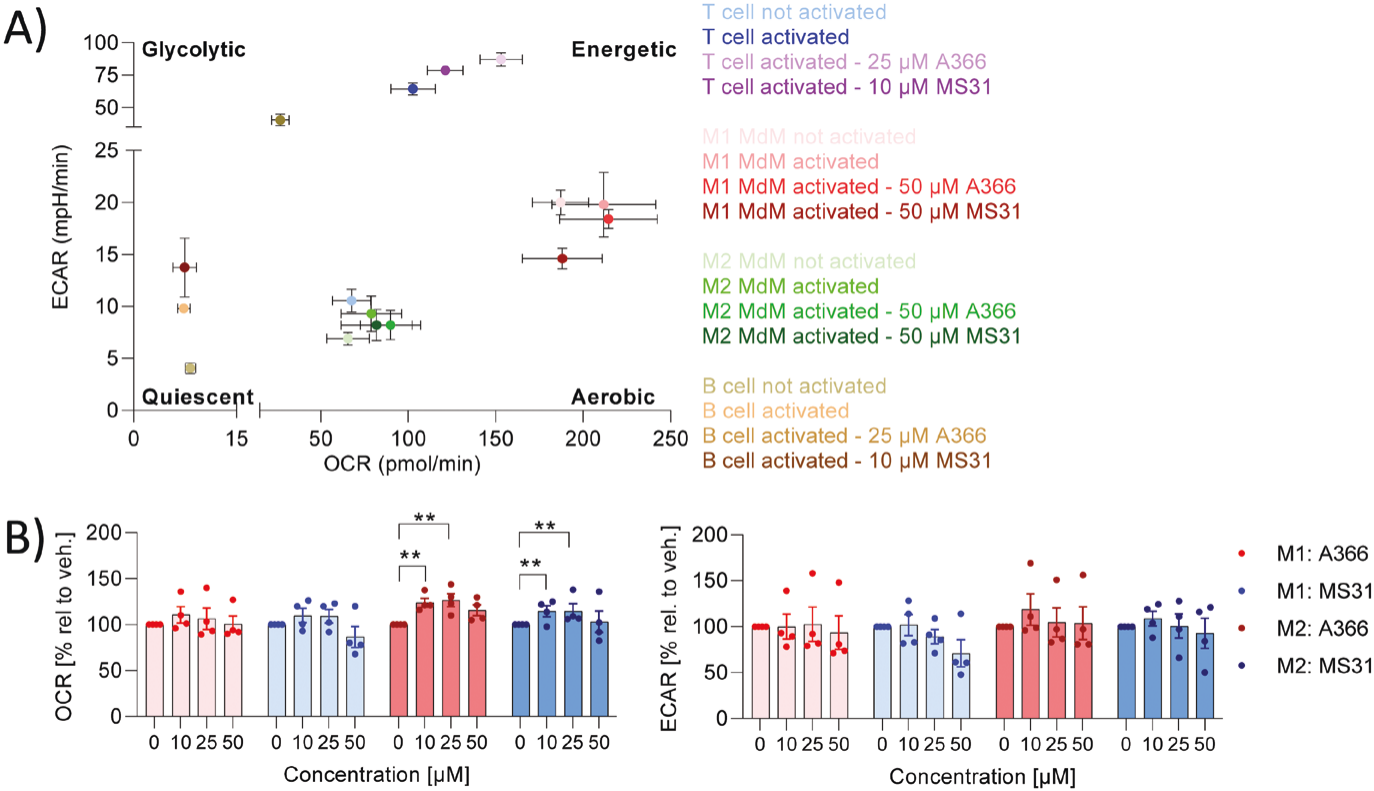
effect of Spindlin-1 modulators on energy metabolism in M1/M2 macrophages, T and B cells. (a) Energy map showing the ECAR and OCR values of various immune cells. (b) Primary human monocytes were differentiated to macrophages and polarized by 10 ng/ml IFNγ or 10 ng/ml IL4 to M1 or M2 macrophages, respectively, in presence or absence of A366 or MS31 with the indicated concentrations for 48 h or 24 h, respectively. (c) CD4^+^ T cells were activated by anti-CD3/anti-CD28 in presence or absence of A366 and MS31 in the indicated concentrations for 5 days. (d) B cells were activated with a stimulation mixture (5 µg/ml anti-IgM, 2.5 µg/ml CpG, 1 µg/ml sCD40L, 50 ng/ml IL-21) for 5 days. Oxidative consumption rate (OCR) and Extracellular acidification rate (ECAR) were determined by Seahorse device. The OCR and ECAR values were normalized to the not activated control sample. *n* = 3. Two-way ANOVA with Dunnett`s multiple comparisons was used to determine significant differences between treated and vehicle (stimulated) samples. *P < 0.05, **P < 0.01, ***P < 0.001.

To meet their energy requirements, M1 macrophages use predominantly glycolysis as an energy source, whereas the main energy source for M2 macrophages is oxidative phosphorylation (Fig. 4a) [19]. Since the Spindlin-1 inhibitors exert some effects on the phenotype of M1 and M2 MdMs, we were interested whether these changes are linked to altered energy metabolism in the macrophages. As a marker for glycolysis, the extracellular acidification rate (ECAR) and as a marker for oxidative phosphorylation the OCR was measured. The OCR for M1-macrophages was about 200 pmol/min and for M2 MdM about 60 pmol/min, whereas the ECAR was approximately 20 mpH/min and 7 mpH/min for M1 MdM and M2 MdM (Fig. 4a), respectively, confirming our previously results [16]. These data indicate that M1 MdMs have a higher energy requirement in comparison to M2 MdMs. A366 and MS31 did not affect the energy metabolism of M1 and only slightly increased OCR in M2 MdMs (Fig. 4a and b).

### Spindlin-1 inhibitors did not affect CD4^+^ T cell activation

**Figure 5:**
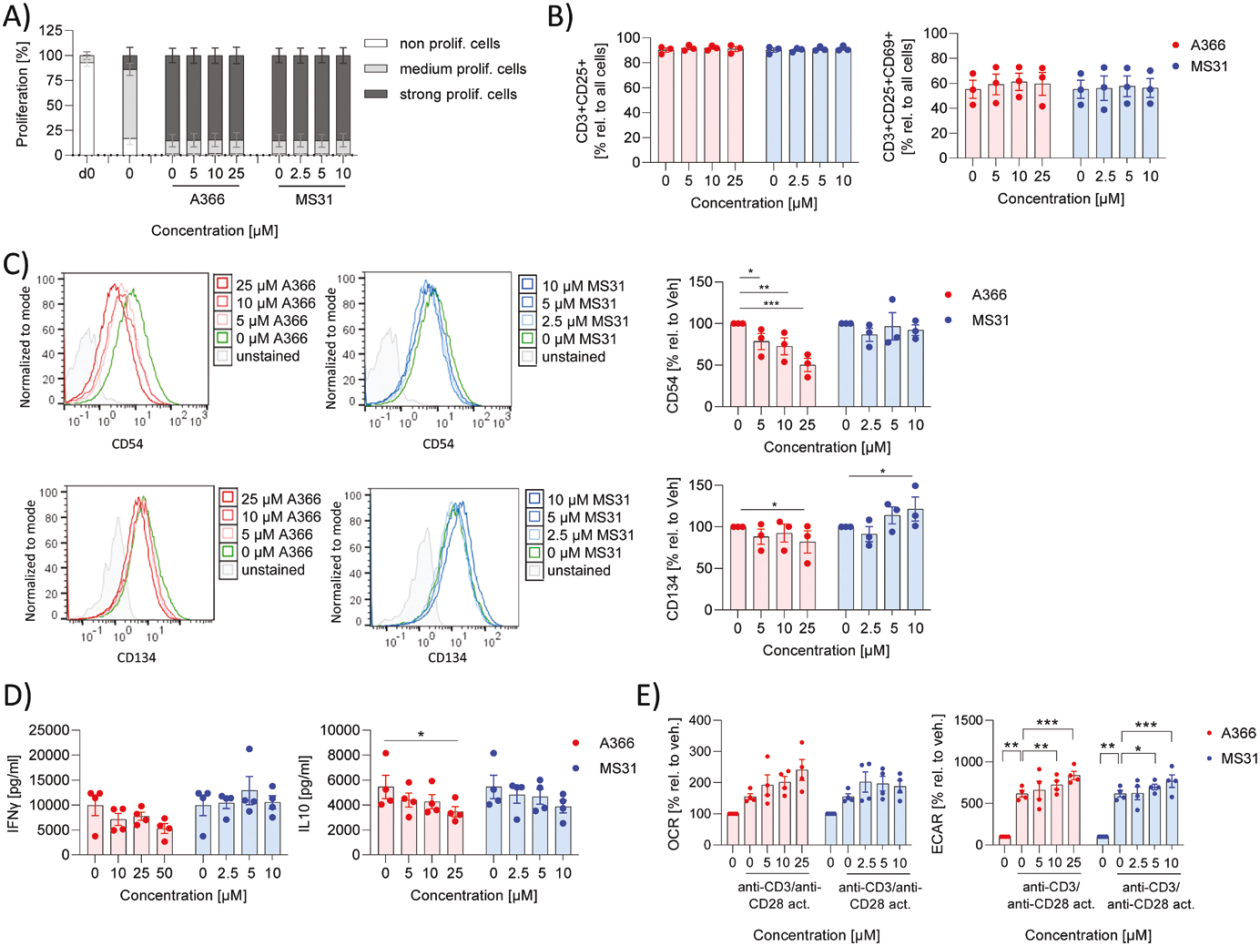
effect of Spindlin-1 modulators on CD4^+^ T cell activation. CD4^+^ T cells isolated from buffy coats by magnetic cell sorting were activated with anti-CD3/anti-CD28 and 50 IU/ml IL-2 in presence or absence of A366 or MS31 in the indicated concentrations for 5 days. (a) For the proliferation assay, CD4^+^ T cells were labeled with the fluorescence day CTV and the MFI was determined by flow cytometry. Using FlowJo Software V10 the cells were gated in fractions with high (nonproliferating cells), medium (medium proliferating cells), and low (strong proliferating c ells) CTV fluorescence. (b) CD3^+^CD25^+^ T cells and CD3^+^CD25^+^CD69^+^ T cells were detected by flow cytometry and analyzed with FlowJo Software V10. (c) Surface markers were determined by flow cytometry. MFI values (left panel) and % values (right panel) are shown. To obtain the % values the MFI values of treated samples were related to vehicle. (d) Cytokines were detected in the supernatant by cytometric bead array. *n* = 3. Two-way ANOVA with Sidak`s multiple comparisons was used to determine significant differences between treated and control samples. *P < 0.05, **P < 0.01, ***P < 0.001.

To address the effect of Spindlin-1 inhibitors on cells of the acquired immune system, the proliferation rate, cytokine release (IL17A, IL10, IFNγ), and surface marker expression (CD69, CD154, CD134, CD25, CD18, CD49a) of CD4^+^ T cells treated with Spindlin-1 inhibitors were analyzed. CD4^+^ T cells were activated with anti-CD3 and anti-CD28. Over the concentration range used, A366 (0–25 µM) and MS31 (0–10 µM) had no cytotoxic effects (Supplementary Fig. 2c). A366 and MS31 did not affect the proliferation of CD4^+^ T cells (Fig. 5a). They did not influence the activation of CD4^+^ T cells, as indicated by the lack of alteration in the number of CD25 positive CD4^+^ T cells and CD25 and CD69 positive CD4^+^ T cells or the activation marker CD69 (Fig. 5b; Supplementary Fig. 5a). A366 reduced the expression of the adhesion molecule ICAM-1 (CD54) and the proliferation/survival marker tumor necrosis factor receptor superfamily 4 (CD134), whereas MS31 increased CD134 expression (Fig. 5c). Adhesion molecules (Integrin beta 2 (CD18), Integrin alpha 1 (CD49a)), and CD40L (CD154) were not influenced by either Spindlin-1 inhibitor (Supplementary Fig. 5a). Spindlin-1 inhibitors had no or only minimal effect on the release of IL10, IL17, and IFNγ (Fig. 5d; Supplementary Fig. 5b). Activation of CD4^+^ T cells leads, as expected, to a significant increase in ECAR (Figs 4a and 5e). Spindlin-1 inhibitors showed only minor effects on energy metabolism. They both shifted CD4^+^ T cells towards the energetic phenotype (Figs 4a and 5e). These data indicate that Spindlin-1 inhibitors may have only minor effects on T-cell function.

### Spindlin-1 inhibitors prevented activation of B cells

**Figure 6:**
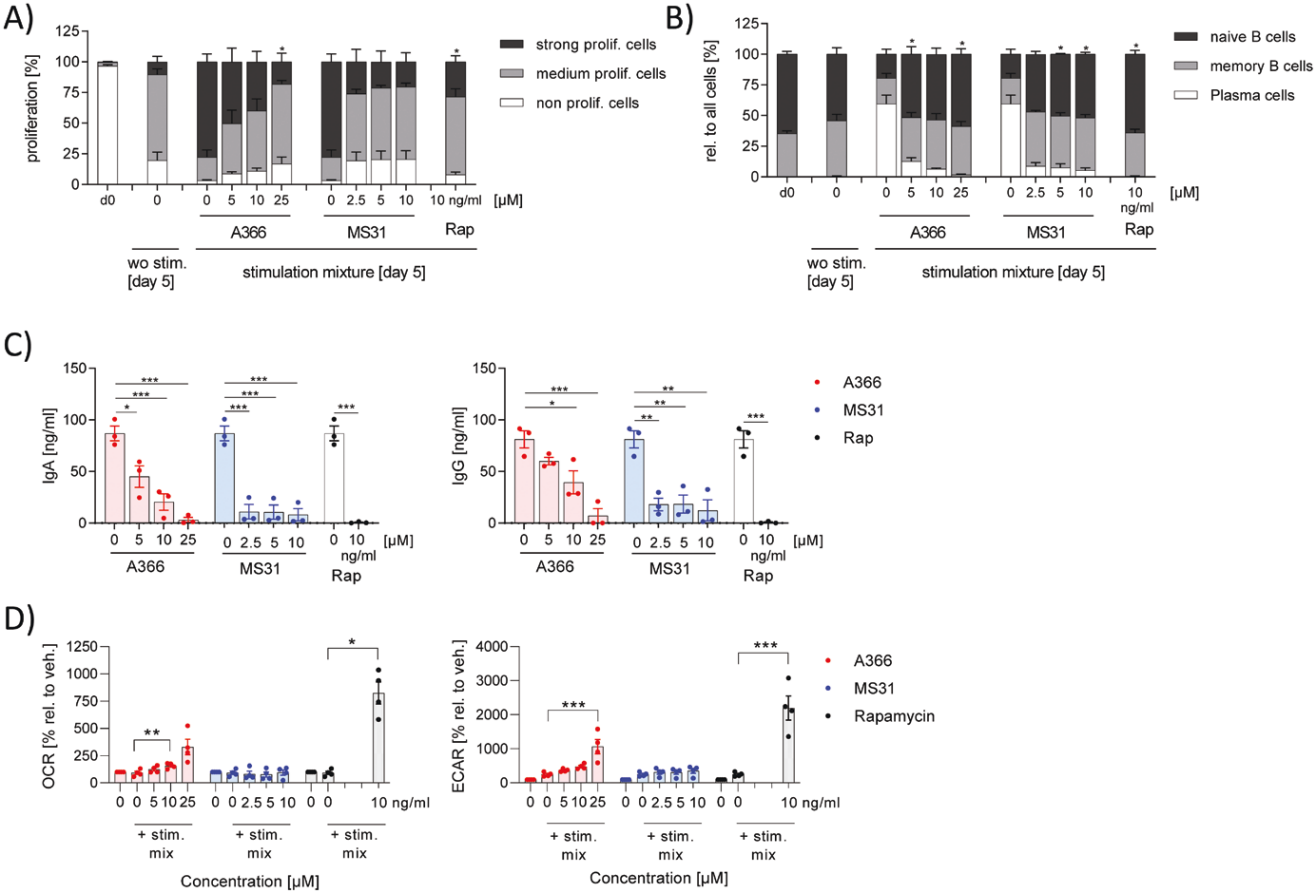
effect of Spindlin-1 modulators on B cell activation. B cells isolated from buffy coats by magnetic cell sorting were activated with 5 µg/ml anti-IgM, 2.5 µg/ml CpG, 1 µg/ml sCD40L, 50 ng/ml IL-21 in presence or absence of A366 or MS31 in the indicated concentrations for 5 days. (a) For the proliferation assay, B cells were labeled with the fluorescence day CTV and the MFI was determined by flow cytometry. Using FlowJo Software V10 the cells were gated in fractions with high (nonproliferating cells), medium (medium proliferating cells), and low (strong proliferating cells) CTV fluorescence. (b) Naïve cells (CD19^+^CD27^low^CD38^med^), memory B cells (CD19^+^CD27^med^CD38^low^), and plasmablasts (CD27^+^CD38^+^) cells were determined by flow cytometry and analyzed by FlowJo Software V10. The number of B cell types were related to all cells. (c) IgG and IgA were determined from the supernatant by ELISA. (d) Oxidative consumption rate (OCR) and Extracellular acidification rate (ECAR) were determined by Seahorse device. The OCR and ECAR values were normalized to the not activated control sample. *n* = 3. Two-way ANOVA with Dunnett`s multiple comparisons was used to determine significant differences between treated and control samples. *P < 0.05, **P < 0.01, ***P < 0.001.

B cells were activated with a mixture of IL-21, sCD40L, CpG, anti-IgM for 5 days in the presence or absence of the Spindlin-1 inhibitors to analyze their effects on B cell function. Spindlin-1 inhibitors, at the concentrations tested, did not induce cytotoxicity (Supplementary Fig. 2d). Activation leads to proliferation of B cells, formation of plasmablasts and the release of immunoglobulins (Ig)G and IgA. As a control substance, rapamycin was used, which was reported to inhibit B cell proliferation, plasmablasts formation and release of IgG and IgA [16] and was confirmed by our results (Fig. 6a–c). Interestingly, A366 and MS31 markedly reduced B cell proliferation, formation of plasmablasts, and the release of IgG and IgA, in a concentration-dependent manner (Fig. 6a–c). The control compound, rapamycin induced both glycolysis and oxidative phosphorylation. Interestingly, only A366 exerted a concentration-dependent increase in energy metabolism (Fig. 6d). Our stimulation mixture included T cell-like activation components (IL-21, sCD40L, anti-IgM) and innate stimulation (CpG). We then evaluated whether T cell-like or innate stimulation is influenced by Spindlin-1 inhibitors. Notably, T cell-like stimulation did not result in induced proliferation, plasmablast formation or release of IgG and IgA (Supplementary Figs 6 and 7). In contrast, CpG stimulation did promote proliferation, plasmablast formation and IgG/IgA release. Proliferation and plasmablast formation were only marginally inhibited, whereas the release of immune globulins was significantly inhibited by the compounds (Supplementary Figs 6 and 7). These data indicate that Spindlin-1 inhibition prevented mainly the innate B cell response.

## Discussion

We studied the impact of Spindlin-1 inhibitors (A366, MS31) on the function of immune cells *in vitro*. We analyzed the effects caused by both inhibitors and attributed these to the inhibition of Spindlin-1. Targeting Spindlin-1 impaired B cell activation, as evidenced by reduced proliferation, plasmablast formation and of IgG/IgA release. Both drugs weakened M2 polarization, indicating a target-specific effect. The effect of MS31 in shifting the M1 towards an M2 phenotype was specific to this compound, as it was not observed with A366. Overall, the inhibition of Spindlin-1 prevented B cell activation may have weakened M2 polarization but had no effect on T cell activation.

The function of Spindlin-1 seems to be manifold. It is responsible for rRNA synthesis [5], it mediates expression of molecules involved in cellular functions such as the cell cycle and adhesion [20, 21]. In HEK293T cells, the presence of Spindlin-1 leads to the accumulation of GDNF, a negative regulator of cell adhesion, resulting in weak cell-to-cell adhesion [21]. However, in primary human endoneural endothelial cells, GDNF induces the expression of the cell adhesion molecule ICAM-1 [22], indicating that inhibition of Spindlin-1 followed by reduced GDNF levels should reduce ICAM-1 expression. In line with this, we observed that inhibition of Spindlin-1 in human primary CD4^+^ T cells resulted in a decrease in the expression of ICAM-1 (CD54). This reduction in ICAM-1 expression may impact the migration potential of CD4^+^ T cells treated with Spindlin-1 inhibitors, leading to a diminished immune response.

Furthermore, we observed that inhibition of Spindlin-1 resulted in a decrease in the formation of plasmablasts. Plasmablasts are the rapidly produced and short-lived effector cells that terminally differentiate into nondividing plasma cells. It has been suggested that epigenetic events occur during the activation of B cells and their transition to plasma cells [23]. Specifically, there are differences in histone marks between quiescent and activated B cells. *In vitro* activation of B cells has been shown to increase methylation of various histone lysines compared to cells at rest [24]. Interestingly, the methylation of H3K4 and H3K9 was found to increase after *in vitro* activation, indicating that these methyl marks are necessary for the transition of B cells to plasma cells [24]. Since Spindlin-1 interacts with these methylated histones, it is likely that the interaction between Spindlin-1 and the methylated histones is crucial for the transition of B cells to plasma cells. Therefore, inhibiting the interaction between methylated histones and Spindlin-1 may prevent the transition of B cells to plasma cells, as observed in our experiments.

On the other hand, the decrease in plasmablast formation observed in the presence of Spindlin-1 inhibitors may be a result of impaired B cell proliferation. Previous studies have already identified a role for Spindlin-1 in the cell cycle. Overexpression of Spindlin-1 in HeLa cells led to an accumulation of cells in the G2/M phase, suggesting that Spindlin-1 interferes with mitosis and causes a halt in proliferation [25]. However, in human seminoma T-Cam2 cells, the opposite effect was found. Overexpression of Spindlin-1 in these cells promoted cell cycle progression, decreasing the number of cells in the G0/G1 phase and increasing those in the S and G2/M phases [20]. Interestingly, we observed an antiproliferative effect of Spindlin-1 inhibitors in B cells, but no effect in CD4^+^ T cells. This suggests that the antiproliferative effect of Spindlin-1 is potentially dependent on the cell type.

A limitation of the study was the inability to ascertain clearly whether the impact of A366 on immune cells stems from its reported modulation of GLP, G9a, or Spindlin-1. This was not feasible as the lowest effective concentration of A366 was 5 µM, significantly higher than the IC_50_ values for G9a (3.3 nM), GLP (38 nM), and Spindlin-1-H3K4me3 interaction (72 nM) [2, 11, 12]. However, cellular activity of A366 in terms of G9a inhibition occurs at 300 nM, and a full reduction of the H3K9me2 signal requires at least 3 days incubation [26]. Moreover, direct binding of A366 to the Tudor domain of Spindlin1 has a KD of 111.1 nM [3]. Additionally, a cellular thermal shift assay with HL-60 cells at 100 µM A366 showed a significant shift in the apparent aggregation temperature compared to the DMSO control, indicating cell penetration and Spindlin1 stabilization through specific A366 binding [3]. These findings suggest that higher concentrations are necessary for A366 to interact with Spindlin1 in a cellular context, which may also explain the higher concentrations needed for immunomodulating effects. To determine whether observed effects are due to Spindlin1 inhibition rather than GLP/G9a, a selective knockout of Spindlin1 might be necessary.

## Conclusion

Our data indicate that the current Spindlin-1 inhibitors strongly interfere with B cell function, suggesting that they can modulate the immune response. Interestingly, CD4^+^ T cells and macrophages, which are the main immune cells that kill tumor cells, were not or only slightly influenced by Spindlin-1 inhibitors. In conclusion, for a full preclinical characterization of anticancer drugs, their interactions with the immune system should be addressed carefully.

## Supplementary Material

uxaf013_suppl_Supplementary_Figures_1-7

## Data Availability

The data that support the findings of this study are available from the corresponding author upon reasonable request.

## Abbreviations

ANOVA: analysis of variance
BSA: bovine serum albumin
CCL: CC-chemokine ligand
CTV: CellTrace^TM^ violet
ECAR: extracellular acidification rate
FCS: fetal calf serum
GDNF: glial-derived neurotrophic factor
GM-CSF: granulocytes macrophage colony-stimulating factor
IFN: interferon
IL: interleukin
MACS: magnetic activated cell sorting
MdM: monocyte-derived macrophages
M-CSF: macrophage colony-stimulating factor
OCR: oxygen consumption rate
PBMCs: human peripheral blood mononuclear cells
PBS: phosphate-buffered saline
RAP: rapamycin
SEM: standard error of the mean
TNF: tumor necrosis factor
