# Supplementary material for: Immune-modulatory effects of Spindlin-1 inhibitors
Source: Clin Exp Immunol. 2025 Jun 13;219(1):uxaf013. doi: 10.1093/cei/uxaf013 (PMC12164290; doi:10.1093/cei/uxaf013)
Supplement: uxaf013_suppl_Supplementary_Figures_1-7 [file uxaf013_suppl_supplementary_figures_1-7.docx]

**Supplemental Material**

**Immune-modulatory effects of Spindlin-1 inhibitors**

Susanne Schiffmann^1,2*^, Marina Henke^1^, Friedemann Weber^3^ and Michael J. Parnham^1,4^

^1^ Fraunhofer Institute for Translational Medicine and Pharmacology ITMP, Theodor-Stern-Kai 7, 60596 Frankfurt am Main, Germany.

^2^ Institute of Clinical Pharmacology, Goethe-University Hospital Frankfurt, Theodor-Stern-Kai 7, 60590 Frankfurt/Main, Germany.

^3^ Institute for Virology, FB10-Veterinary Medicine, Justus-Liebig University, 35392 Giessen, Germany

^4^ Current address: EpiEndo Pharmaceuticals ehf, Bjargargata 1, 102 Reykjavik, Iceland

**Supplemental Figure 1:** Gating Strategy and Dotblots for B cell activation. B cells isolated from buffy coats by magnetic cell sorting were activated with a stimulation mixture consisting of 5 µg/ml anti IgM, 2.5 µg/ml CpG, 1 µg/ml sCD40L, 50 ng/ml IL-21 in presence or absence of 25 μM A366 or 10 μM MS31 for 5 days. A) Gating strategy for B cells (day 0) using FlowJo Software V10. B) One representative blot for samples at day 5 without stimulation, with stimulation + Vehicle, with stimulation + 25 μM A366 and with stimulation + 10 μM MS31. Naïve cells (CD19+CD27lowCD38med), memory B cells (CD19+CD27medCD38low) and plasmablasts (CD27+CD38+) cells were determined.


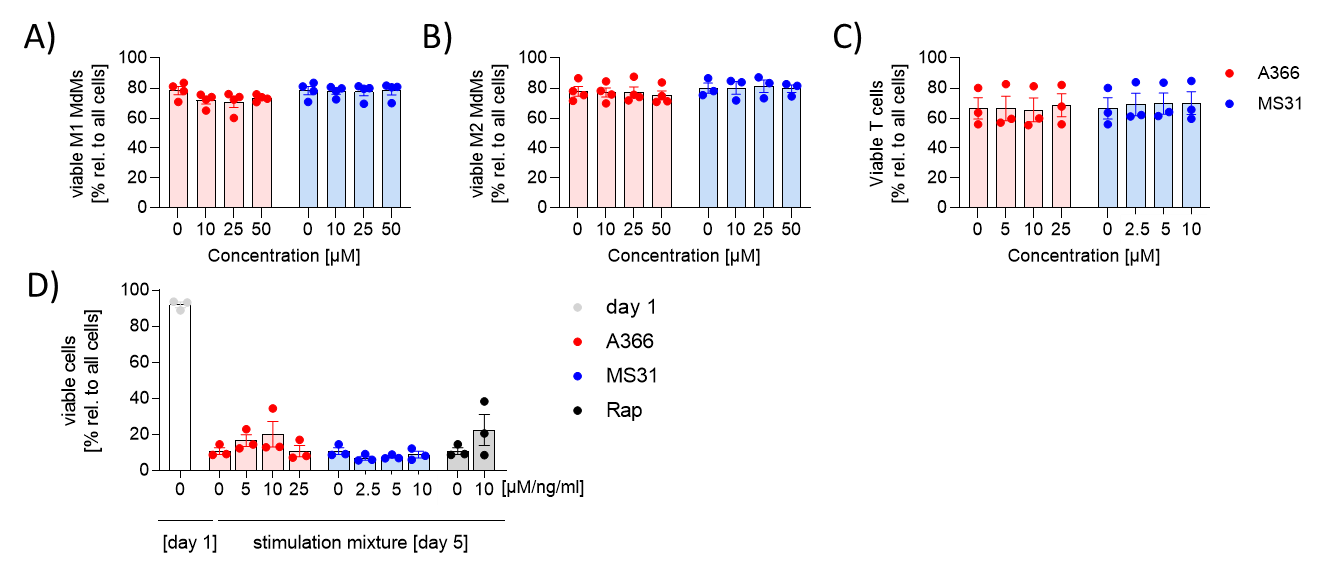


**Supplemental Figure 2:** Effect of Spindlin-1 modulators on viability. A/B) Monocytes isolated from buffy coats by magnetic cell sorting were differentiated to M1 MdMs (A) or M2 MdMs (B) by the addition of 10 ng/ml GM-CSF or 10 ng/ml M-CSF, respectively. Macrophages were polarized by 10 ng/ml IFNγ to M1 or by 10 ng/ml IL4 to M2 macrophages in presence or absence of A366 (10, 25, 50 μM), MS31 (10, 25, 50 μM) or vehicle for 48h. C) T cells isolated from buffy coats by magnetic cell sorting were cultured with 50 IU/ml IL2 in presence of A366 (5, 10, 25 μM), MS31 (2.5, 5, 10 μM) or vehicle for 5 days. D) B cells isolated from buffy coats by magnetic cell sorting were cultured with 5 µg/ml anti IgM, 2.5 µg/ml CpG, 1 µg/ml sCD40L, 50 ng/ml IL-21 in presence of A366 (5, 10, 25 μM), MS31 (2.5, 5, 10 μM), 10 ng/ml Rapamycin or vehicle for 5 days. Apoptosis was determined by zombie staining. Number of viable cells were related to all cells. n = 3-4. Two-way ANOVA with Dunnett`s multiple comparisons was used to determine significant differences between treated and control samples.


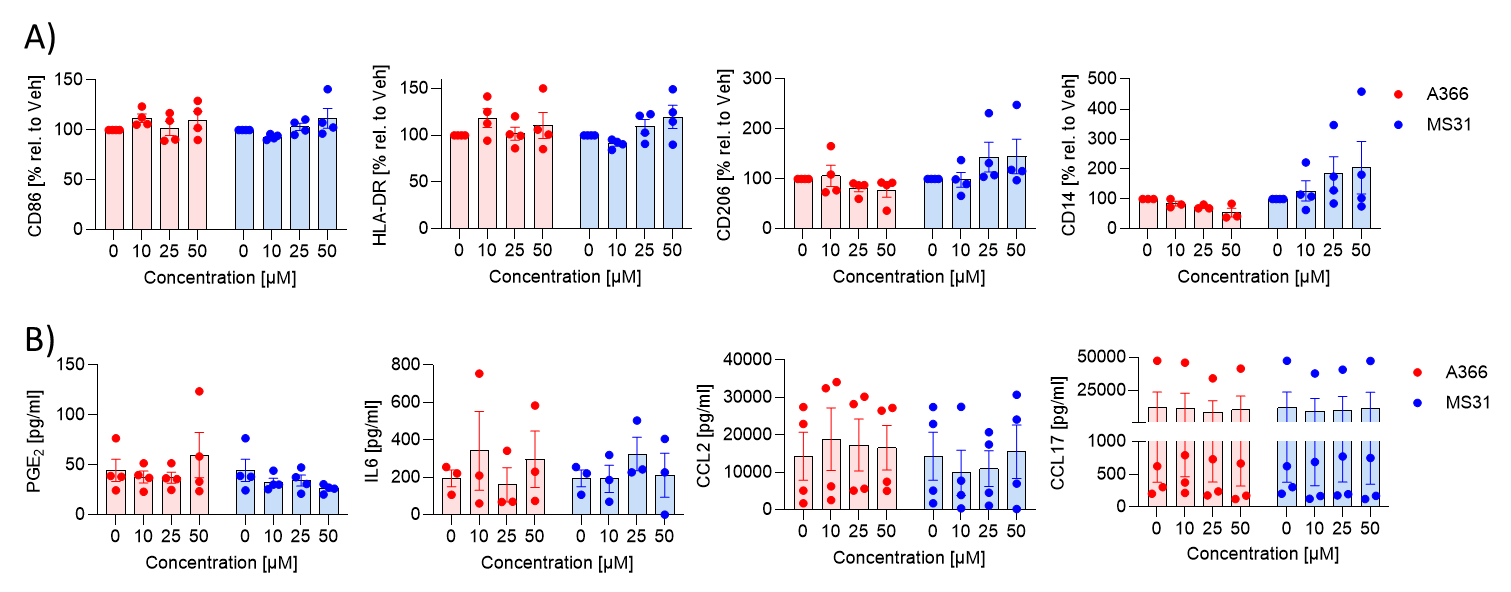


**Supplemental Figure 3:** Effect of Spindlin-1 modulators on M1 polarization. Monocytes isolated from buffy coats by magnetic cell sorting were differentiated to macrophages by the addition of 10 ng/ml GM-CSF. Macrophages were polarized by 10 ng/ml IFNγ to M1 macrophages in presence or absence of A366 or MS31 in the indicated concentrations for 48h. A) Surface markers were determined by flow cytometry. The MFI values of treated samples were related to vehicle. B) Cytokines were determined by cytometric bead array or ELISA in the supernatant. n = 3-4. Two-way ANOVA with Dunnett`s multiple comparisons was used to determine significant differences between treated and control samples.


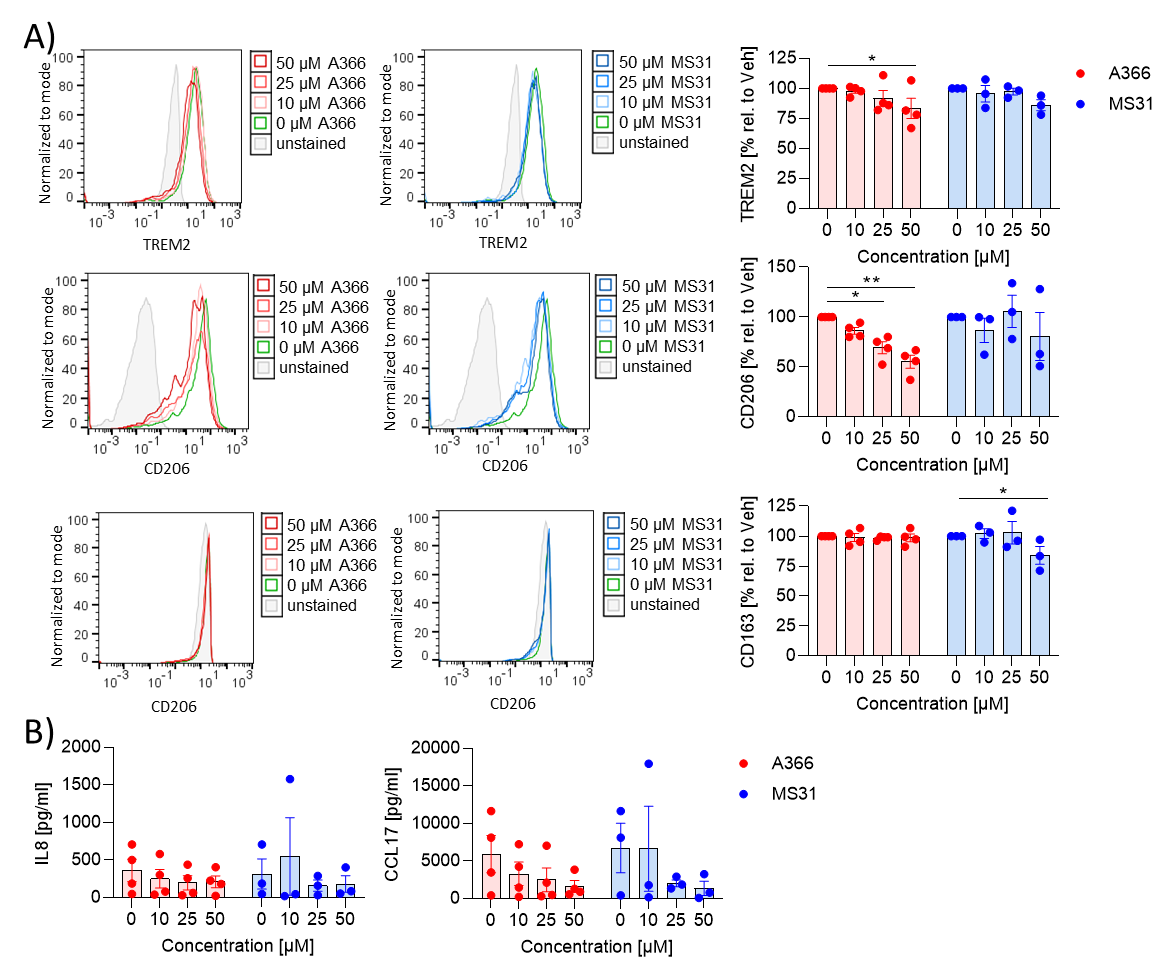


**Supplemental Figure 4:** Effect of Spindlin-1 modulators on M2 polarization. Monocytes isolated from buffy coats by magnetic cell sorting were differentiated to macrophages by the addition of 10 ng/ml M-CSF. Macrophages were polarized by10 ng/ml IL4 to M2 macrophages in presence or absence of A366 or MS31 in the indicated concentrations for 24h. A) Surface markers were determined by flow cytometry. The MFI values of treated samples were related to vehicle. One representative histogram of surface markers is shown. C) Cytokines were detected by cytometric bead array or ELISA in the supernatant. n = 2-3. Two-way ANOVA with Dunnett`s multiple comparisons was used to determine significant differences between treated and control samples. *p<0.05, **p<0.01.

**Supplemental Figure 5:** Effect of Spindlin-1 modulators on T cell activation. T cells isolated from buffy coats by magnetic cell sorting were activated with CD3/CD28 and 50 IU/ml IL-2 in presence or absence of A366 or MS31 in the indicated concentrations for 5 days. A) Surface markers were determined by flow cytometry. The MFI values of treated samples were related to vehicle. B) IL-17 was detected by cytometric bead array in the supernatant. n = 3. Two-way ANOVA with Sidak`s multiple comparisons was used to determine significant differences between treated and control samples.

**Supplemental Figure 6:** Effect of Spindlin-1 modulators on B cell activation. B cells isolated from buffy coats by magnetic cell sorting were activated with a T cell like stimulation (5 µg/ml anti IgM, 1 µg/ml sCD40L, 50 ng/ml IL-21) or innate stimulation (2.5 µg/ml CpG) in presence or absence of A366, MS31 or rifampicin in the indicated concentrations for 5 days. A) For the proliferation assay, B cells were labeled with the fluorescence day CTV and the MFI was determined by flow cytometry. Using FlowJo Software V10 the cells were gated in fractions with high (non proliferating cells), medium (medium proliferating cells) and low (strong proliferating cells) CTV fluorescence. B) Naïve cells (CD19+CD27lowCD38med), memory B cells (CD19+CD27medCD38low) and plasmablasts (CD27+CD38+) cells were determined by flow cytometry and analyzed by FlowJo Software V10. The number of B cell types were related to all cells. n = 3. Two-way ANOVA with Dunnett`s multiple comparisons was used to determine significant differences between treated and control samples.

**Supplemental Figure 7:** Effect of Spindlin-1 modulators on the release of IgG and IgA of activated B cells. B cells isolated from buffy coats by magnetic cell sorting were activated with a combination of T cell like and innate (A/D), a T cell like (B/E) (5 µg/ml anti IgM, 1 µg/ml sCD40L, 50 ng/ml IL-21) or innate (C/F) stimulation (2.5 µg/ml CpG) in presence or absence of A366, MS31 or rifampicin in the indicated concentrations for 5 days. IgA (A-C) and IgG (D-F) level were determined from the supernatant by ELISA. n = 3. Two-way ANOVA with Dunnett`s multiple comparisons (test substances) or t-test (Rifampicin) was used to determine significant differences between treated and control samples.
